# Endothelial damage in major depression patients is modulated by SSRI treatment, as demonstrated by circulating biomarkers and an *in vitro* cell model

**DOI:** 10.1038/tp.2016.156

**Published:** 2016-09-06

**Authors:** I Lopez-Vilchez, M Diaz-Ricart, V Navarro, S Torramade, J Zamorano-Leon, A Lopez-Farre, A M Galan, C Gasto, G Escolar

**Affiliations:** 1 Department of Hemotherapy and Hemostasis, Hospital Clinic of Barcelona, Biomedical Diagnosis Centre, Institute of Biomedical Research August Pi i Sunyer, University of Barcelona, Barcelona, Spain; 2 Department of Psychiatry, Hospital Clinic of Barcelona, Institute Clinic of Neurosciences, Barcelona, Spain; 3 Department of Medicine, School of Medicine, Complutense University, Madrid, Spain

## Abstract

There is a link between depression, cardiovascular events and inflammation. We have explored this connection through endothelial dysfunction, using *in vivo* and *in vitro* approaches. We evaluated circulating biomarkers of endothelial dysfunction in patients with major depression at their diagnosis (MD-0) and during antidepressant treatment with the selective serotonin reuptake inhibitor escitalopram, for 8 and 24 weeks (MD-8 and MD-24). Results were always compared with matched healthy controls (CON). We measured *in vivo* circulating endothelial cells (CECs) and endothelial progenitor cells (EPCs) in blood samples, and assessed plasma levels of soluble von Willebrand factor (VWF) and vascular cell adhesion molecule-1 (VCAM-1). CEC counts, soluble VWF and VCAM-1 were statistically elevated in MD-0 (*P*<0.01 versus CON) and gradually decreased during treatment. Conversely, EPC levels were lower in MD-0, tending to increase throughout treatment. *In vitro* studies were performed in human endothelial cells cultured in the presence of sera from each study group. Elevated expression of the inflammation marker intercellular adhesion molecule-1 and oxidative stress, with lower presence of endothelial nitric oxide synthase and higher reactive oxygen species production, were found in cells exposed to MD-0 sera (*P*<0.05 versus CON). These results were normalized in cells exposed to MD-24 sera. Thrombogenicity of extracellular matrices generated by these cells, measured as expression of VWF, tissue factor and platelet reactivity, showed non-significant differences. We provide a model of cultured endothelial cells reproducing endothelial dysfunction in naive patients with major depression, demonstrating endothelial damage and inflammation at diagnosis, and recovering with selective serotonin reuptake inhibitor treatment for 24 weeks.

## Introduction

Cardiovascular disease and depression are two comorbid conditions with high prevalence in developed countries, and constitute an important health concern.^1, 2^ According to experts from the American Heart Association, depression should join the ranks of obesity, diabetes, hypertension and smoking as an official heart disease risk factor.^2^ Depression is commonly present in patients with coronary heart disease and is independently associated with increased cardiovascular morbidity and mortality.^2^ Moreover, the effect of antidepressant drugs on cardiac risk in some epidemiological studies indicate that patients with major depression treated with selective serotonin reuptake inhibitors (SSRIs) had reduced cardiovascular risk compared with patients not receiving antidepressant therapy.^3, 4^ There is evidence of a link between depression, cardiovascular events and inflammation, although the precise mechanisms involved remain unclear.^5^

A growing body of evidence indicates that endothelial dysfunction is associated with multiple clinical conditions with high cardiovascular risk, including depression.^6, 7^ Endothelial dysfunction is characterized by an alteration of the response of the endothelium towards reduced vasodilation, closely dependent on nitric oxide (NO) levels.^8^ There is an inverse correlation between depressed mood and endothelial function, which can be measured by noninvasive methods such as flow-mediated dilation.^9^ An alternative approach to assess endothelial dysfunction is through measurement of plasma levels of endothelium-related markers, which can provide information on overall endothelial activation or dysfunction. A recent publication summarizing the findings of the Maastricht Study supports the concept of an association between endothelial dysfunction and depression from a more biochemical point-of-view.^10, 11^

Endothelial damage and activation is characterized by increased plasma levels of soluble fractions of intercellular adhesion molecule-1 (ICAM-1), vascular cell adhesion molecule-1 (VCAM-1) and von Willebrand factor (VWF) that are considered surrogate markers of the presence, severity and risk of vascular disease.^12, 13^

Detachment of endothelial cells from the vascular wall is another characteristic of endothelial damage, leaving a disrupted endothelium and facilitating platelets to interact with the exposed subendothelium, thus promoting their activation. Presence of increased circulating endothelial cells (CEC) is associated with endothelial damage.^14^ Conversely, reduced endothelial progenitor cells (EPCs) have been found in pathological conditions that concur with endothelial dysfunction, such as acute cardiovascular events, uremia or diabetes.^15, 16, 17^ In this regard, there is recent evidence on reduced levels of circulating EPCs in patients with depression and acute coronary syndromes.^18, 19^ Oxidative stress, measured by circulating markers (superoxide dismutase, glutathione, oxLDL, among others) in plasma and platelets,^10^ vascular relaxation^20^ and animal models,^21, 22^ has been closely associated with major depression.^23, 24^ Studies in a depression model with chronic mild stress rats demonstrated associations between endothelial dysfunction, oxidative stress and depression-like symptoms that were modulated by chronic SSRI.^25^

Despite the existing evidence, the direct damaging effects on the endothelium of the humoral changes occurring in patients with depression remains poorly explored. In previous studies, we thoroughly characterized endothelial dysfunction in different pathologic entities such as uremia, obesity, stem cell transplant, acute myocardial infarction and stroke,^15, 26, 27^ using both the analysis of *ex vivo* biomarkers and a well-characterized *in vitro* model with cultured endothelial cells. The present study was designed with two major goals: to analyze the profile of circulating markers of endothelial damage present in blood from patients with major depression and to elucidate the direct effect of the patients' sera on the endothelium by using the *in vitro* cell model. In addition, we assessed the potential modulating effect of antidepressant treatment with the SSRI escitalopram during 24 weeks.

## Materials and methods

### Patients

The study was approved by our institution Ethics Committee (2009-4964). Written informed consent was obtained from patients and healthy volunteers. Twelve patients diagnosed with major depression and 12 matched healthy individuals as controls (CON) were included in the study. Patients were studied at the moment of diagnosis and during treatment with the SSRI escitalopram (SCIT) for 8 weeks (MD-8), when patients start to show first mood improvements, and for 24 weeks (MD-24), when patients showed signs equivalent to remission of the depressive symptoms.

Patients with major depression were diagnosed and evaluated by the Department of Psychiatry at our institution. Out-patients aged between 18 and 65 years with unipolar depression were recruited. To be eligible for inclusion, patients had to fulfill the DSM-IV criteria for a current depressive episode and to have had a baseline score in the Spanish version of the 17-item Hamilton Rating Depression Scale (HDRS) of >17.^28^ Exclusion criteria included any history of mania, hypomania or non-affective psychosis, and current substance dependence. Comorbidity with medical conditions related to cardiovascular risk, such as established atherosclerotic vascular disease, coronary syndrome, stroke, obesity (body mass index ≥30 kg/m^2^), diabetes, uncontrolled blood pressure or low-density lipoprotein-cholesterol and current smoking (>10 cigarettes per day) was considered an exclusion criteria. A non-compliance with the previous conditions resulted in the exclusion from the study. To ensure the treatment compliance, escitalopram plasma levels were assessed in 4-, 8- and 24-week follow-up visits. A negative result in at least one assessment was also considered as an exclusion criteria. Clinical assessment of depressive symptoms was rated on every visit. Matched CON were assessed by the same criteria to discard any subjacent affective disorder. Participants in the study were not allowed to take drugs affecting hemostasis (non-steroidal anti-inflammatory drugs, aspirin, among others) within the 10 days prior to collection of blood samples.

Baseline blood determinations were performed in patients and CON at time of recruitment (MD-0). Immediately after, patients were started on oral escitalopram as antidepressant therapy. The initial dose was established at 5 mg per day and maintained for 4 days. Dose was increased up to 10 mg per day on the fifth day and adjusted by the attending psychiatrist as required until a maximum of 40 mg per day. Blood determinations were repeated in patients at 8 (MD-8) and 24 (MD-24) weeks during treatment. The average doses of escitalopram used in our studies was 28 mg per day.

### Blood sampling and serum collection

Blood samples were drawn from the antecubital vein and collected into different anticoagulants attending to the experimental purposes. Blood in sodium heparin was used in the assessment of CECs and EPC. Serum samples to supplement the endothelial cell-culture media were obtained from tubes without anticoagulant. Determinations of soluble biomarkers of endothelial damage were performed in citrated plasma samples.

Plasma and sera samples were obtained by centrifugation of the tubes at 1030*g* for 15 min, divided into aliquots and immediately stored at −20°C or −70°C until analysis.

### Measurement of CECs and EPCs

Flow cytometry was applied to measure CECs defined as CD45^−^ CD146^+^ CD31^+^, and EPCs defined as CD45^−^ CD34^+^ KDR^+^.^16^ Samples were processed, in duplicate, within 4 h after blood extraction. Two 150 μl blood samples were incubated with 10 μl of the corresponding monoclonal antibodies; PE anti-CD146, FITC anti-CD31 and PerCP anti-CD45 for CEC measurements, and with FITC anti-CD34, PerCP anti-CD45 and PE anti-kinase-insert domain receptor (KDR) for EPC measurements. Red blood cells in the samples were lysed for 15 min with BD FACS Lysing Solution. Samples were then centrifuged, washed twice, resuspended in PBS with LMWH (50 U ml^−1^) and analyzed using a FACScan flow cytometer (Becton Dickinson, Madrid, Spain). Cells stained with isotopic CON for IgG conjugated with the different fluorochromes were used as negative CON. Assessment of 2 × 10^6^ events per sample was considered sufficient for statistical analysis. Acquisition files were analyzed with the Kaluza Flow Cytometry Analysis software version 1.2.12286.11234 (Beckman Coulter, Brea, CA, USA). Cell numbers were calculated by multiplying the ratio of CEC and EPC obtained in the flow cytometry analysis by the number of leukocytes per ml in the blood sample measured in an Advia 2120 Hematology System (Siemens, Deerfield, IL, USA), and levels were expressed as the absolute number of both CEC and EPC per 1 ml of whole blood.

### Assessment of soluble markers of endothelial damage

Plasma levels of VWF and soluble VCAM-1 (sVCAM-1) were analyzed by using the commercially available kits DG-EIA VWF activity kit (Diagnostic Grifols, Barcelona, Spain) and Human sVCAM-1 ELISA (Chemicon-Millipore, Temecula, CA, USA), respectively. All measurements were performed in duplicate and concentrations determined from the standard curve according to the manufacturer's instructions. Levels of soluble VWF were expressed as % of VWF in plasma samples, and levels of sVCAM-1 were expressed as ng per ml plasma.

### Endothelial cell-culture model and isolation of the extracellular matrices

Endothelial cells were isolated from human umbilical cord veins by collagenase A treatment (0.2% in phosphate buffered saline (PBS) 15 min, 37 °C), according to a previously described method.^29^ Cells were grown with MEM 199 culture medium, supplemented with 100 U ml^−1^ penicillin, 50 mg ml^−1^ streptomycin and 20% pooled human serum. Human umbilical vein endothelial cells (HUVECs) were grown at 37 °C in a 5% CO_2_ humidified incubator. The culture medium was changed every 48 h. After the second passage, endothelial cells were subcultured from 3 to 7 days on 0.1% gelatin-coated coverslips contained in 6-well plates, in the presence of 20% pooled human serum corresponding to each studied condition, 0, 8 and 24 weeks of antidepressant treatment or healthy individuals, grouping samples into four different pools. Incubation times depended on the cell density required for immunofluorescence studies, 2–3 days for studies in cell and 6–7 days to generate a proper extracellular matrix. Extracellular matrices were isolated with incubation of cell cultures with 4% EGTA (pH 7.40), to detach cells without damaging the subendothelial layer.

### Production of intracellular ROS

Reactive oxygen species (ROS) generation, increased with the oxidative stress, was explored by using the cell-permeable ROS detection reagent CM-H_2_DCFDA (5-(and 6)-chloromethyl-2′,7′-dichlorodihydrofluorescein diacetate acetyl ester).^29^ Cultured endothelial cells seeded on six-well plates, were incubated with 10 μm CM-H2DCFDA (37 °C for 15 min). Cells were washed three times with PBS followed by cell stimulation with the same culture media containing 20% sera at the different time points studied, for 30 min. Intracellular ROS production was monitored in a fluorescence Leica DM4000B microscope (Leica Microsystems, Wetzlar, Germany). Up to 15–20 micrographs were taken from each condition with a Leica DFC310FX camera (Leica Microsystems, Wetzlar, Germany) and the Leica Application Suite (v.3.8.0) software (Leica Microsystems, Heerbrugg, Switzerland).

### Immunofluorescence studies in HUVEC and extracellular matrices

Expression of ICAM-1 was evaluated on the surface of cultured endothelial cells, as inflammation marker. Intracellular endothelial NO synthase (eNOS) was explored to assess the oxidative state of cultured HUVEC, as expression of this enzyme is sensitive to oxidative stress. Modifications in the presence of the main adhesive and procoagulant proteins, VWF and tissue factor (TF), on the extracellular matrices generated by cultured cells were also investigated as thrombogenic markers.

Fixed endothelial cell cultures and extracellular matrices were processed for a two-step immunofluorescence as previously described.^29^ Coverslips were first incubated with 0.1% glycine-PBS to inactivate aldehyde groups and blocked with 1% BSA-PBS. Antibodies to ICAM-1, eNOS, VWF and TF were detected with the corresponding secondary antibodies conjugated with different fluorochromes (Alexa Fluo −555, −488 and −594, respectively). Samples were mounted in prolong with antifade reagent and observed in a Leica DM4000B microscope. Up to 15–20 micrographs were taken from each condition with a Leica DFC310FX camera and the Leica Application Suite (v.3.8.0) software. The density of labeling was calculated by computerized morphometric analysis.

### Densitometry analysis of fluorescence

Fluorescence micrographs were densitometrically analyzed using the Image J software (v.1.43 m) (Rasband, W.S., Image J, National Institutes of Health, Bethesda, MD, USA, http://rsb.info.nih.gov/nih-image/manual/tech.html#analyze). This software automatically analyses the gray density of each pixel in a scale ranging from 0 (black) to 255 (white), in the corresponding fluorescence channel. Cultured cells were selected from the background with the threshold tool, and the fluorescence intensity was measured only in the selected area. Immunofluorescence results were expressed as fold-increase in the mean fluorescence intensity (mean gray value) versus the healthy CON value.

### Thrombogenicity of extracellular matrices generated by HUVECs in flow studies

The thrombogenicity of the ECMs generated was assessed in perfusion studies using parallel-plate perfusion chambers, in which coverslips coated with the extracellular matrices generated by cultured cells were used as thrombogenic substrata for circulating blood.^26^ Twenty two milliliters of citrated blood samples from healthy individuals were recirculated using a peristaltic pump through the perfusion chamber adjusted to reach a shear rate equivalent to 600 s^−1^, for 5 min. After perfusion, surfaces were rinsed with PBS (0.15 m), fixed with 0.5% glutaraldehyde (in 0.15 m PBS) at 4 °C for 24 h and stained with toluidine blue 0.05% for morphometric *en face* evaluation.

Images from the perfused surfaces were obtained using a Leica DFC310FX camera adapted to a Leica DM4000B microscope and the Leica Application Suite (v. 3.8.0) software. Platelet coverage in each micrograph was analyzed using the Image J software (v.1.43 m). Results were expressed as mean percentage of surface covered by platelets (%CS) versus the total area screened in each studied condition.

### Statistics

Data are expressed as mean±s.e.m., and Student's *t*-test for paired data was used for comparisons between two different conditions after normalities of the distributions were confirmed through Kolmogorov–Smirnov test. The minimal level of statistical significance was established at *P*<0.05. Statistical measurements were performed with the STATGRAPHICS Plus for Windows 4.1 software (Statistical Graphics, Rockville, MD, USA).

### Reagents

Monoclonal antibodies to determine CECs and EPCs were PE-conjugated anti-CD146 (Clone S-Endo1; Biocytex, Marseille, France; Cat# 5050-PE100T), FITC-conjugated anti-CD31 (BD Pharmingen, San Diego, CA, USA; Cat# 555445), PerCP-conjugated anti-CD45 (2D1; Becton Dickinson, San Jose, CA, USA; Cat# 345809), FITC-conjugated anti-CD34 (8G12; Becton Dickinson, San Jose, CA, USA; Cat# 345801) and PE-conjugated anti-hVEGF R2/KDR (IgG1; R&D Systems, Minneapolis, MN, USA; Cat.# FAB357P). BD FACS Lysing Solution was from Becton Dickinson (San Jose, CA, USA; Cat# 349202). Low molecular weight heparin (LMWH) was Fragmin (Pharmacia & Upjohn, Stockholm, Sweden). DG-EIA vWF activity kit (Diagnostic Grifols; Cat# 218008). Human sVCAM-1 ELISA kit (Chemicon-Millipore; Cat# ECM340). Reagents used in HUVEC cultures were: Cell culture six-well plates (TPP, Trasadingen, Switzerland; Cat# 92006). Collagenase (Boehringer Mannheim, Mannheim, Germany; Cat# 10103586001); culture medium (MEM 199; Gibco BRL, Life Technologies, Scotland, UK; Cat# 31150-030); penicilin/streptomycin (Cat# 15140-122) and trypsin-EDTA (Cat# 59418C-100 ML) were from Gibco (Life Technologies, Grand Island, NY, USA). ROS detection reagent CM-H_2_DCFDA was from Molecular Probes (by Life Technologies; Eugene, OR, USA; Cat# C6827). Immunofluorescence antibodies were: Mouse anti-human ICAM-1 ((P2A4); Millipore; Cat# MAB2146); rabbit anti-human NO synthase (eNos (C-20); Santa Cruz Biotechnology, Dallas, TX, USA; Cat# sc-654); rabbit anti-human VWF (Dako, Glostrup, Denmark; Cat# A0082) and mouse anti-human TF (American Diagnostica, Stanford, CT, USA; Cat# 4509); secondary antibodies conjugated with either Alexa Fluo-488 (Cat# A11017), -555 (Cat# A31570) or -594 (Cat# A11012) were from Invitrogen-Molecular Probes (Eugene, OR, USA). Mounting media was ProLong Gold antifade reagent from Molecular Probes (by Life Tecnologies, Eugene, OR, USA; Cat# P36934).

## Results

### Description of the groups

We collected samples from CON and MD-0 patients (HDRS value of 26.5±4.2 versus 0.59±1.2 in CON, *P*<0.001), aged between 18 and 65 years, without statistical significance in both groups attending age or sex. Severity of depressive symptoms gradually diminished with antidepressant treatment for 8 and 24 weeks, as demonstrated the decreasing evolution of the HDRS scores at these time points (*P*<0.001 versus MD-0). A more detailed description of the patients included is summarized in Table 1.

### CECs and EPCs

CEC were significantly higher in blood from MD-0 patients than in CON (*P*<0.01 versus CON). Treatment with a SSRI gradually decreased CEC levels, reaching values after 24 weeks equivalent to those found in samples from healthy individuals (*P*<0.05 in MD-24 versus MD-0 and MD-8). These data paralleled with the improvement on the depressive symptoms measured by the HDRS score (Table 1).

**Figure 1:**
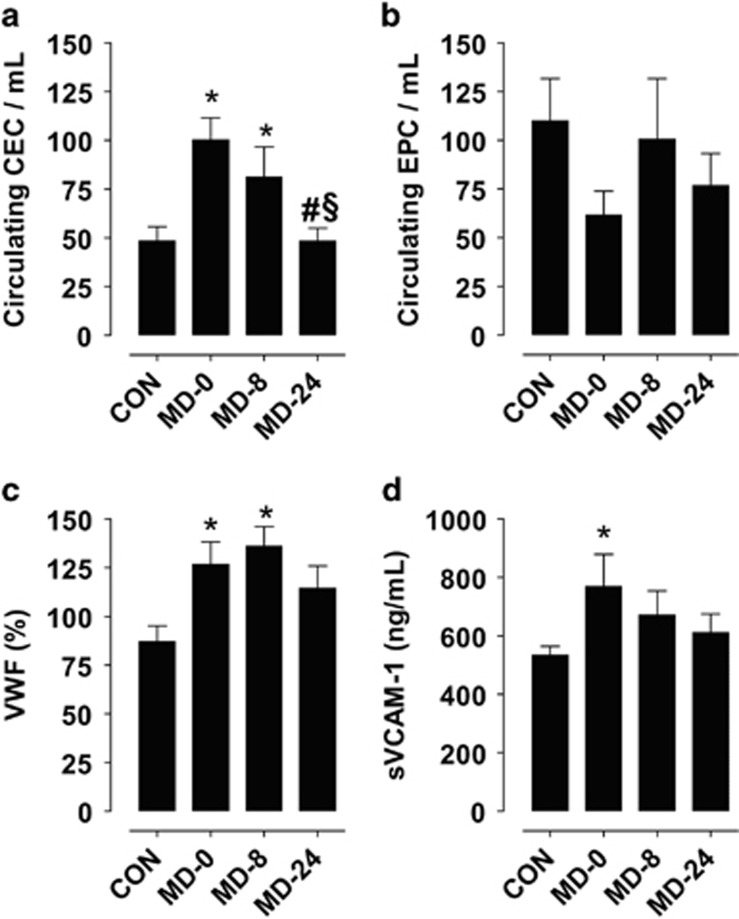
Markers of endothelial damage in blood samples. Bar diagrams showing *in vivo* levels of markers of endothelial damage: circulating endothelial cells (CEC) and endothelial progenitor cells (EPC) in whole blood samples (**a, b**), and von Willebrand factor (VWF) and soluble vascular cell adhesion molecule-1 (sVCAM-1) in plasma samples (**c**, **d**). Graphs show levels of these markers in healthy controls (CON) and in major depression patients at the moment of diagnosis (MD-0), and after 8 and 24 weeks of antidepressant treatment (MD-8 and MD-24) with the selective serotonin reuptake inhibitor escitalopram. Results are expressed as mean±s.e.m. (*n*=12). *P<0.01 versus CON; ^#^*P*<0.05 versus MD-0; ^§^*P*<0.05 versus MD-8.

On the contrary, levels of EPC were lower in MD-0 patients than in CON blood samples, showing a tendency to increase in MD-8 and MD-24. However, differences did not reach statistical significance and levels were not totally normalized after 24 weeks of antidepressant treatment. Results are summarized in Figures 1a and b.

### Presence of soluble markers of endothelial damage in blood samples

When analyzing soluble VWF and VCAM-1 in plasma, we found statistically significant increased levels of both markers in samples from MD-0 patients with respect to CON plasma samples (*P*<0.01). Treatment with a SSRI for 24 weeks reduced the soluble levels of these markers. However, while sVCAM-1 levels seemed to reduce more efficiently throughout treatment, VWF showed a delayed response since levels remained unchanged after 8 weeks of treatment. Results are summarized in Figures 1c and d.

### Oxidative state in cultured endothelial cells exposed to patients' sera

**Figure 2:**
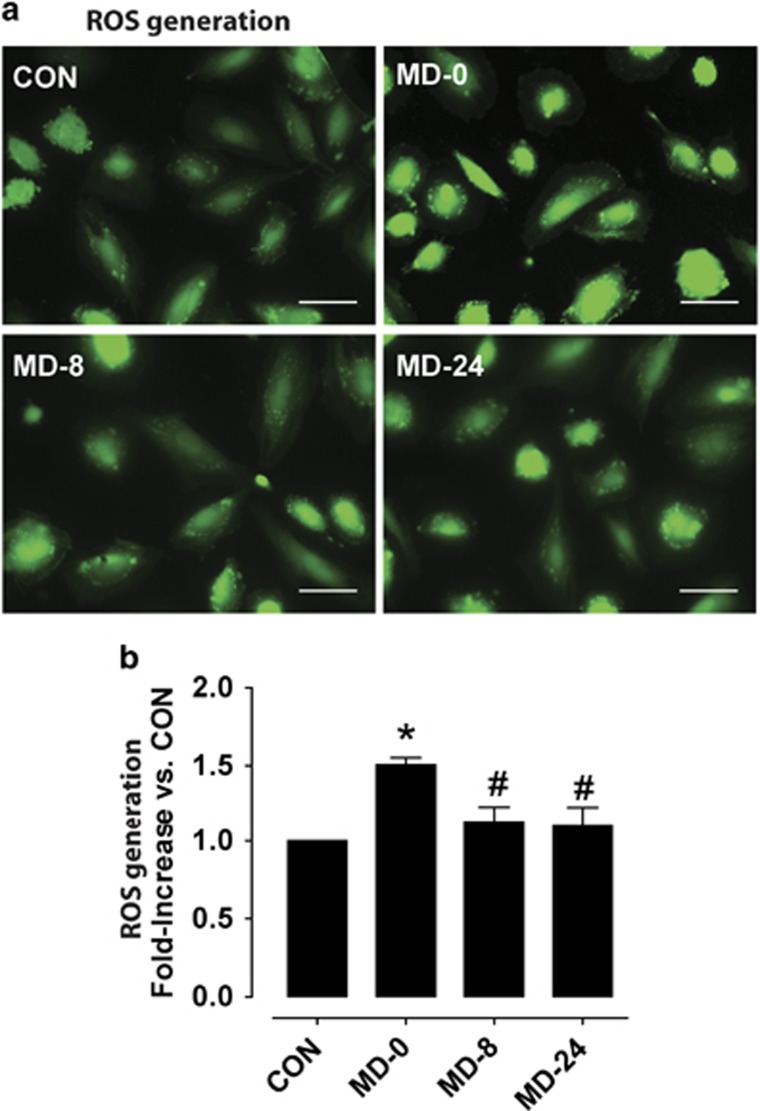
Production of reactive oxygen species (ROS) in cultured endothelial cells. (**a**) Representative fluorescence micrographs showing *in vitro* ROS production in cells grown with cell-culture media supplemented with sera from: healthy controls (CON) and major depression patients at the moment of diagnosis (MD-0), and after 8 and 24 weeks of antidepressant treatment (MD-8 and MD-24) with the selective serotonin reuptake inhibitor escitalopram (scale bar, 50 μm). (**b**) Bar diagrams summarizing quantification of fluorescence intensities, as fold-increase in the mean fluorescence intensity in each condition over the mean fluorescence measured in CON. Results are expressed as mean±s.e.m. (*n*=4 sera pools). *P<0.05 versus CON, ^#^*P*<0.05 versus MD-0.

Cultured endothelial cells exposed to cell-culture media supplemented with 20% of patients' sera, induced a statistically significant increase in intracellular ROS production in cells exposed to sera from patients at the moment of diagnosis in comparison to CON sera (*P*<0.01). ROS production appeared quickly restored during treatment with the SSRI escitalopram (*P*<0.05 in MD-8 and MD-24 versus MD-0). Results are summarized in Figure 2.

**Figure 3:**
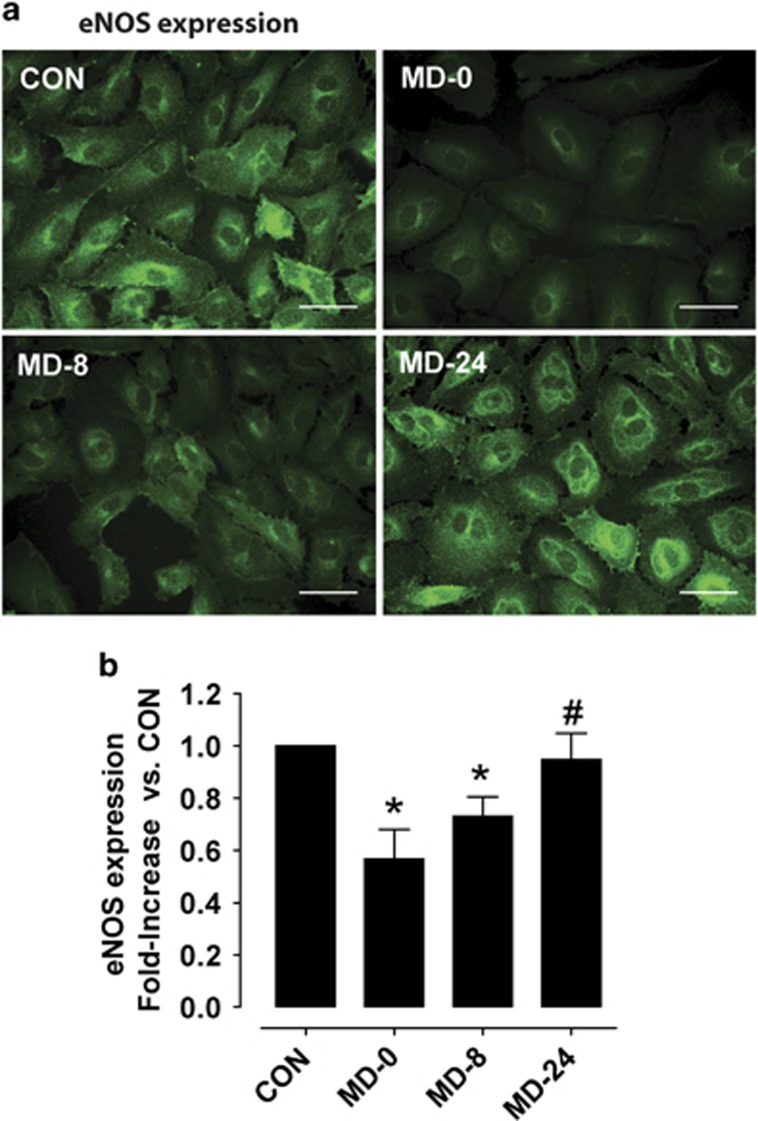
Expression of the oxidative stress marker eNOS within cultured endothelial cells. (**a**) Representative fluorescence micrographs showing *in vitro* intracellular eNOS in cells grown with cell-culture media supplemented with sera from: healthy controls (CON) and major depression patients at the moment of diagnosis (MD-0), and after 8 and 24 weeks of antidepressant treatment (MD-8 and MD-24) with the selective serotonin reuptake inhibitor escitalopram (scale bar, 50 μm). (**b**) Bar diagrams summarizing quantification of fluorescence intensities, as fold-increase in the mean fluorescence intensity in each condition over the mean fluorescence measured in CON. Results are expressed as mean±s.e.m. (*n*=4 sera pools). *P<0.05 versus CON, ^#^*P*<0.05 versus MD-0. eNOS, endothelial nitric oxide synthase.

In contrast, assessment of eNOS by immunofluorescence showed the opposite tendency, with a diminished expression in cells exposed to MD-0 sera versus CON (*P*<0.01), that was gradually and statistically normalized during antidepressant treatment, reaching levels similar to those in CON samples. See Figure 3 for more detail.

### Expression of inflammation markers on the surface of cultured endothelial cells

**Figure 4:**
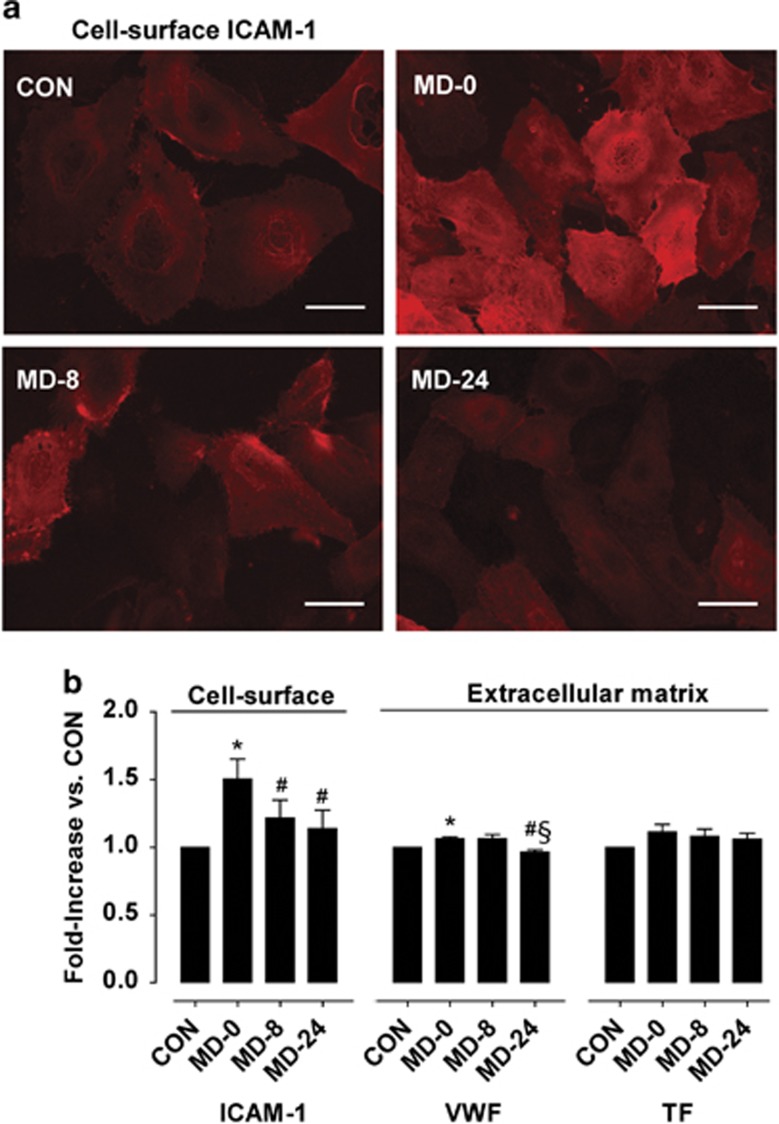
Expression of the inflammation marker intercellular adhesion molecule-1 (ICAM-1) on the surface of cultured endothelial cells and thrombogenicity of extracellular matrices generated by these cultured endothelial cells. (**a**) Representative fluorescence micrographs showing *in vitro* expression of ICAM-1 in the surface of endothelial cells grown with cell-culture media supplemented with sera from: healthy controls (CON) and major depression patients at the moment of diagnosis (MD-0), and after 8 and 24 weeks of antidepressant treatment (MD-8 and MD-24) with the selective serotonin reuptake inhibitor escitalopram (scale bar, 50 μm). (**b**) From left to right, bar diagrams summarizing quantification of fluorescence intensities from immunofluorescence studies showing ICAM-1 on the cell surface, and presence of VWF and tissue factor (TF) in extracellular matrices, expressed as fold-increase in the mean fluorescence intensity in each condition over the mean fluorescence measured in CON. Results are expressed as mean±s.e.m. (*n*=4 sera pools). *P<0.05 versus CON, ^#^*P*<0.05 versus MD-0, ^§^*P*<0.05 versus MD-8. VWF, von Willebrand factor.

As summarized in Figure 4, cultured endothelial cells exposed to sera from the patients at the moment of diagnosis, showed a statistically significant increase in the expression of ICAM-1 at the cell surface (*P*<0.01 versus CON). When cells were incubated with sera from the same patients under treatment with escitalopram, there was a rapid reduction in the expression of ICAM-1, which was notable even 8 weeks after initiating this treatment (*P*<0.05 versus MD-0) and showed after 24 weeks levels almost comparable to those measured in healthy individuals (*P*<0.05 versus MD-0).

### Thrombogenicity of extracellular matrices generated by cultured endothelial cells

Modifications in the presence of the main adhesive and procoagulant proteins, VWF and TF, on the extracellular matrices were evaluated by immunofluorescence (expressed as fold-increase versus CON) and studies under flow conditions. Changes in the levels of VWF on the extracellular matrices paralleled those found in plasma samples, being slightly but statistically higher on extracellular matrices produced by cells exposed to sera from MD-0 patients than from healthy CON (*P*<0.05). Expression of VWF remained unchanged on extracellular matrices from cells incubated with sera from MD-8 patients, and decreased statistically on extracellular matrices exposed to sera from MD-24 patients (*P*<0.05 versus MD-0 and MD-8).

TF expression appeared moderately higher on extracellular matrices produced by cells exposed to sera from MD-0 patients with respect to levels on extracellular matrices with CON sera and significantly decreased in a gradual manner on extracellular matrices produced by cells exposed to sera from patients under treatment with escitalopram for 8 and 24 weeks.

The reactivity towards platelets of the extracellular matrices produced by HUVEC cultured in the presence of the sera under study (CON, MD-0, MD-8 and MD-24) was assessed in adhesion studies under flow conditions. After perfusing the extracellular matrices with citrated blood, we did not find differences in the percentage of surface covered by platelets between either of the different treatment periods or healthy CON (% platelet coverage: 19.9±1.1, 19.2±1.3, 20.5±1.0 and 18.3±0.5; corresponding to CON, MD-0, MD-8 and MD-24, respectively).

## Discussion

Inflammation and endothelial damage are mechanisms potentially connecting depression to cardiovascular disease.^5, 30, 31^ Results from the present study demonstrate significant elevation of different plasma markers of endothelial activation and damage in patients with major depression. Moreover, treatment with a SSRI shows a protective role in the modulation of the endothelial damage developed in these patients. In addition, these findings were experimentally reproduced in endothelial cells cultured in the presence of sera from patients with major depression.

Our present data demonstrate endothelial activation and damage in our study group in comparison with healthy individuals. We observed increased levels of plasma biomarkers, such as soluble VCAM-1, VWF and circulating CEC, and decreased number of circulating EPC, at diagnosis. Increased levels of soluble VCAM-1 and VWF have been reported in other studies in major depression,^10, 32, 33^ and support the existence of endothelial damage and cardiovascular risk. Reduced EPC counts have been found in pathological conditions that concur with endothelial dysfunction, including patients with depression with and without acute coronary syndromes.^15, 17, 34, 35, 36^ In addition, presence of increased CECs is associated with endothelial damage.^14^ Our study confirms that this pattern is also present in our patients. Interestingly, measured levels of CECs appeared significantly elevated especially in association with the highest HDRS scores (≥16), which correspond to greater depressive symptoms. In previous studies, we reported a similar pattern of circulating markers in pathologies with an associated cardiovascular risk such as uremia, diabetes, obesity, myocardial infarction and stroke.^15, 26, 27, 37, 38^

Our present study, has further explored the potential modulating effect of the antidepressant treatment with the SSRI escitalopram, during 24 weeks. Our results show significant reductions in soluble VCAM-1 and VWF levels and in CEC counts during treatment with escitalopram. Moreover, there was also a tendency to increase the number of EPC, a finding that has been considered as an indicator of vascular repair. Our results regarding soluble levels of VWF are in agreement with those in a recent publication on the serum proteomic profile in major depression patients.^33^ Authors reported elevated VWF levels in patients with a current episode, and normalized in patients with a remitted depressive episode. However, a previous study by Dome *et al.*^36^ described elevated levels of circulating EPC in major depression patients, but they did not find modifications in this parameter during recovery of the depressive episode with antidepressant treatment; which could be due to the short follow-up (4 weeks).

In addition to the *ex vivo* measurements, we were able to reproduce *in vitro* the endothelial activation and damage associated with major depression; by incubating cultured endothelial cells in the presence of sera from the patients included. Exposure to sera collected at the moment of diagnosis (MD-0) caused activation of endothelial cells, as measured by increases of ICAM-1, and oxidative stress with the production of ROS and lower presence of eNOS when compared with cells exposed to sera from healthy individuals. However, no relevant modifications were detected in the thrombogenicity of extracellular matrices generated by these cells, which appeared moderately enriched in VWF and TF. Altogether, cells exposed to sera from patients at the moment of major depression diagnosis displayed a proinflammatory phenotype, accompanied with a state of oxidative stress. Interestingly, this pathologic profile was gradually prevented in those cells previously incubated with sera from patients during antidepressant treatment with escitalopram for 8 and 24 weeks (MD-8 and MD-24), showing profiles more similar to those with sera from healthy CON.

Endothelial dysfunction is characterized by a shift in the actions of the endothelium towards reduced vasodilation, closely dependent on NO levels. There is growing evidence during the last decade indicating an association of oxidative stress with major depression.^23, 24^ In the present study, we have confirmed increased rates of endothelial activation and oxidative stress in major depression at the moment of the initial diagnosis. Furthermore, we found a protective effect of escitalopram as shown by the gradual restoration of ROS production and intracellular eNOS expression at 8 and 24 weeks. These results are in agreement with the CREATE study, designed to evaluate whether citalopram treatment yielded additional antiplatelet benefit in depressed patients with coronary artery disease, where they found improved endothelial function after treatment with citalopram for 12 weeks, as derived by an enhanced production of NO.^39^ Our *in vitro* studies also agree with those by Eren *et al.*,^22^ in which authors described that experimental depression in a model with chronic mild stress rats was associated with elevated oxidative stress and that treatment with escitalopram for 4 weeks provided a protective effect. Matchkov *et al.*,^25^ also reported associations between endothelial dysfunction, oxidative stress and depression-like symptoms that were modulated by chronic SSRI using the same model of depression. Connection between oxidative stress and major depression patients was also demonstrated by Sarandol *et al.*^40^ Studies from the same group confirmed that oxidative stress in major depression patients was partially reversed after 24 weeks of antidepressant treatment.^41^ A recent meta-analysis by Jimenez-Fernandez *et al.*^42^ indicates that oxidative stress plays a key role in depression and that antidepressant activity of some drugs could be partially explained through their antioxidant action.

Despite the relative increases in the presence of TF and VWF in the extracellular matrices generated by cultured endothelial cells exposed to MD-0 sera, studies using flowing blood resulted in similar reactivity towards platelets, which remained unaltered throughout antidepressant treatment (MD-8 and MD-24). Platelets are involved in the development and precipitation of cardiovascular events, and are also the main carriers of serotonin.^43^ Serotonin has complex effects on the cardiovascular system that seem to be regulated by the vascular endothelium. Experimental studies from our group have demonstrated that exogenous addition of serotonin potentiates the procoagulant behavior of platelets and enhances the thrombus formation on damaged vascular surfaces, and that these effects are modulated by the presence of SSRIs.^44, 45^ Using the same experimental approach, we have also recently reported a marked procoagulant profile (thrombus and fibrin formation) in blood samples from major depression patients at the moment of diagnosis, which was normalized during antidepressant treatment with the SSRI escitalopram, for 24 weeks.^46^ Therefore, our previous and present results demonstrate that there are two hemostatic components whose responses may be altered in major depression: platelets and the endothelium, with the possibility of both acting synergistically.^47^ Our findings differ from those described by Dawood *et al.*,^48^ in which they reported both vascular function and platelet reactivity within the normal range in major depression patients prior to treatment with SSRI for 12 weeks. However, our data will still be in agreement with the CREATE study that demonstrated beneficial effects of antidepressant treatment in those markers of endothelial damage found previously altered in patients with major depression.^39^

In conclusion, results from the present study suggest that patients with major depression at the moment of diagnosis present alterations compatible with a moderate endothelial dysfunction. The characteristics of the endothelial dysfunction improves with treatment and parallels the clinical evolution of the depressive symptoms evaluated through the HDRS score. Alteration in endothelial markers and responses may not totally explain the thrombogenicity usually associated with major depression patients by itself, but may still contribute to it together with other humoral factors, oxidative stress, inflammation and serotonergic mechanisms. Antidepressant treatment with SSRI for 24 weeks normalized the alterations in the previous biomarkers and improved indicators of inflammation and oxidative stress analyzed in cultured endothelial cells, reinforcing the evidence of a protective vascular effect in patients with major depression. We provide a cell model with cultured endothelial cells capable of reproducing endothelial activation, inflammation and oxidative stress in major depression, which may be useful to test new therapeutic approaches aimed at counteracting the endothelial dysfunction that develops in these patients.

## Figures and Tables

**Table 1:** Demographic and clinical characteristics of patient samples

*Variable*	*Value (*n*=12)*
Sex (% men)	41.7
Age at first episode (years)	46.5±9.8
Number of previous episodes	0.4±0.7
	
*Index episode*	
Age (years)	49.6±10.5
Duration of episode (months)	5.0±2.1
MD-0 HDRS score	26.5±4.2^#^
MD-8 HDRS score	9.1±5.2*^#^
MD-24 HDRS score	3.2±4.2*^#^
	
*Healthy controls*	
Sex (% men)	41.7
Age (years)	48.0±8.5
HDRS score	0.59±1.2

Abbreviations: CON, controls; HDRS score, Hamilton Depression Rating Scale-17 items; MD-0, major depression patients at the moment of diagnosis; MD-8 and MD-24, major depression patients during antidepressant treatment with escitalopram for 8 and 24 weeks

Values are expressed as mean±s.d. (*n*=12). *P<0.001 versus MD-0, ^#^*P*<0.05 versus CON.
